# Tumor-associated neutrophils in lung adenocarcinoma: from mechanisms to therapeutic targeting

**DOI:** 10.3389/fimmu.2026.1892405

**Published:** 2026-07-20

**Authors:** Ruichen Zhang

**Affiliations:** School of Clinical and Basic Medicine, Shandong First Medical University & Shandong Academy of Medical Science, Jinan, China

**Keywords:** immunotherapy resistance, lung adenocarcinoma, N1/N2 polarization, neutrophil extracellular traps (NET), targeted therapy, tumor-associated neutrophils

## Abstract

Lung adenocarcinoma (LUAD) is the predominant subtype of non-small cell lung cancer (NSCLC), characterized by a complex tumor microenvironment (TME) where tumor-associated neutrophils (TANs) exert dual functions—a concept now well-established in broader NSCLC and pan-cancer studies. Despite the advances in immune checkpoint inhibitors (ICIs), primary and acquired resistance remain major obstacles. This review comprehensively dissects the regulatory network of TANs in LUAD, including their recruitment via the CXCR1/CXCR2 axis, polarization towards anti-tumorigenic N1 or pro-tumorigenic N2 phenotypes under the influence of TGF-β and IFN-γ, and the formation of neutrophil extracellular traps (NETs). We highlight the dual functions of TANs and NETs in both tumor promotion (immunosuppression, angiogenesis, metastasis) and tumor suppression (direct cytotoxicity, antigen presentation). Furthermore, we evaluate current therapeutic strategies targeting TANs, such as CXCR1/2 inhibitors (SX-682, Navarixin), PAD4 inhibitors (Cl-amidine), NET-degrading enzymes (DNase I), TGF-β inhibitors (Galunisertib), and myeloid modulators, with a focus on LUAD-specific preclinical and clinical evidence. Major challenges, including off-target toxicity, infection risk, and the lack of predictive biomarkers, are discussed. By bridging mechanistic insights with translational opportunities, this review provides a framework for developing neutrophil-targeted combination therapies to overcome immunotherapy resistance in LUAD.

## 1 Introduction

Lung cancer remains the leading cause of cancer-related morbidity and mortality worldwide (1, 2). Many patients are diagnosed at an advanced stage due to the lack of early symptoms (3). Smoking remains the predominant risk factor (4). Non-small cell lung cancer (NSCLC) accounts for approximately 80% to 85% of all lung cancer cases, among which lung adenocarcinoma (LUAD) is the most common histological subtype (5, 6). Although immune checkpoint inhibitors (ICIs) have significantly improved the prognosis of advanced NSCLC, the objective response rate in LUAD remains only 20%–30%, and primary or acquired resistance is common (6, 7). The contribution of non-T-cell components within the tumor microenvironment (TME), particularly myeloid cells, to immunotherapy resistance is increasingly recognized (8).

Neutrophils are the first line of defense against microbial infections, constituting 50% to 70% of circulating leukocytes in humans (9). Traditionally viewed as short-lived, terminally differentiated effector cells involved solely in acute inflammation, recent studies have revealed that neutrophils exhibit remarkable heterogeneity and functional plasticity within the TME (10, 11). Fridlender et al. first proposed a seminal, albeit now simplified, framework using mouse models, classifying tumor-associated neutrophils (TANs) into anti-tumorigenic N1 and pro-tumorigenic N2 phenotypes, where transforming growth factor β (TGF-β) is a key driver of N2 polarization (12). Although this model laid an important foundation for subsequent research, single-cell studies have since revealed that TANs actually exist along a continuous functional spectrum, with phenotypes far more complex than this dichotomy (13, 14). N1-like neutrophils exhibit high expression of tumor necrosis factor α (TNF-α), intercellular adhesion molecule 1 (ICAM-1), and Fas, exerting anti-tumor effects via reactive oxygen species (ROS) and apoptosis induction. In contrast, N2-like neutrophils highly express arginase 1 (ARG1), CCL2, and CXCL1, promoting T-cell suppression, angiogenesis, and tumor cell proliferation (12, 15). Although this binary classification provides a conceptual framework, the markers for human N1/N2 TANs are not universally established, and their functional status is dynamically regulated by tumor type, stage, and microenvironmental signals, as confirmed in human lung cancer studies (16, 17). Importantly, beyond polarization, neutrophils can also form neutrophil extracellular traps (NETs)—web-like structures composed of decondensed chromatin and granule proteins (e.g., myeloperoxidase, neutrophil elastase)—which not only capture pathogens but also promote cancer metastasis, thrombosis, and immune evasion (18, 19).

Current reviews on TANs in lung cancer largely focus on pan-cancer contexts or NSCLC as a whole, lacking a systematic summary of LUAD-specific neutrophil regulation (20, 21). Different lung cancer subtypes exhibit distinct immune microenvironment features: single-cell transcriptomic analyses reveal that LUAD harbors higher proportions of dendritic cells and monocytes/macrophages compared with lung squamous cell carcinoma, and exhibits more active crosstalk between tumor cells and immune cell populations, suggesting that, theoretically, LUAD may be more amenable to immunotherapy (8). However, the mechanisms of TAN recruitment, polarization, NETosis, and their interactions with T cells and macrophages in LUAD remain to be fully elucidated. Furthermore, existing studies often focus on single molecules or cell types, lacking a multi-dimensional framework that integrates molecular mechanisms with clinical translation.

This review aims to systematically integrate the regulatory network of TANs within the LUAD microenvironment, focusing on their recruitment and polarization mechanisms, the dual functions of NETs, their interactions with other immune cells, and current therapeutic strategies targeting TANs (including CXCR2 inhibitors, PAD4 inhibitors, and NET degradation), supported by preclinical and clinical evidence. By connecting mechanistic research with clinical translation, we provide a theoretical basis for comprehensive neutrophil-targeted immunotherapy in LUAD and identify directions for future translational research.

## 2 Basic characteristics of TANs in LUAD

### 2.1 Origin and recruitment of TANs

Neutrophils originate from hematopoietic stem cells in the bone marrow, differentiating through common myeloid progenitors and granulocyte-monocyte progenitors, and mature under the regulation of granulocyte colony-stimulating factor (G-CSF) and granulocyte-macrophage colony-stimulating factor (GM-CSF) before being released into circulation (22, 23). Mature neutrophils highly express the chemokine receptors CXCR1 and CXCR2, whose ligands include CXCL1, CXCL2, CXCL5, CXCL6, and CXCL8. In the NSCLC (including LUAD) microenvironment, tumor cells and stromal cells abundantly secrete these chemokines, recruiting circulating neutrophils to tumor sites via the CXCR1/CXCR2 axis, forming TANs (24–26). Single-cell RNA sequencing has further revealed the heterogeneity of TANs in NSCLC (including LUAD), with subpopulations exhibiting distinct gene expression profiles, such as a CD66b+ mature subpopulation, an interferon-stimulated gene-expressing subpopulation, a “hybrid” subpopulation with antigen-presenting features, and a pro-angiogenic subpopulation (13, 27). Notably, due to the low mRNA content and fragile structure of neutrophils, conventional droplet-based single-cell sequencing often underestimates their proportion; high-sensitivity platforms such as BD Rhapsody can more accurately capture the TAN lineage, confirming that TANs account for 10%–20% of immune cells in the NSCLC TME (13).

### 2.2 Conceptual evolution of TAN polarization: from N1/N2 dichotomy to functional spectrum

**Figure 1:**
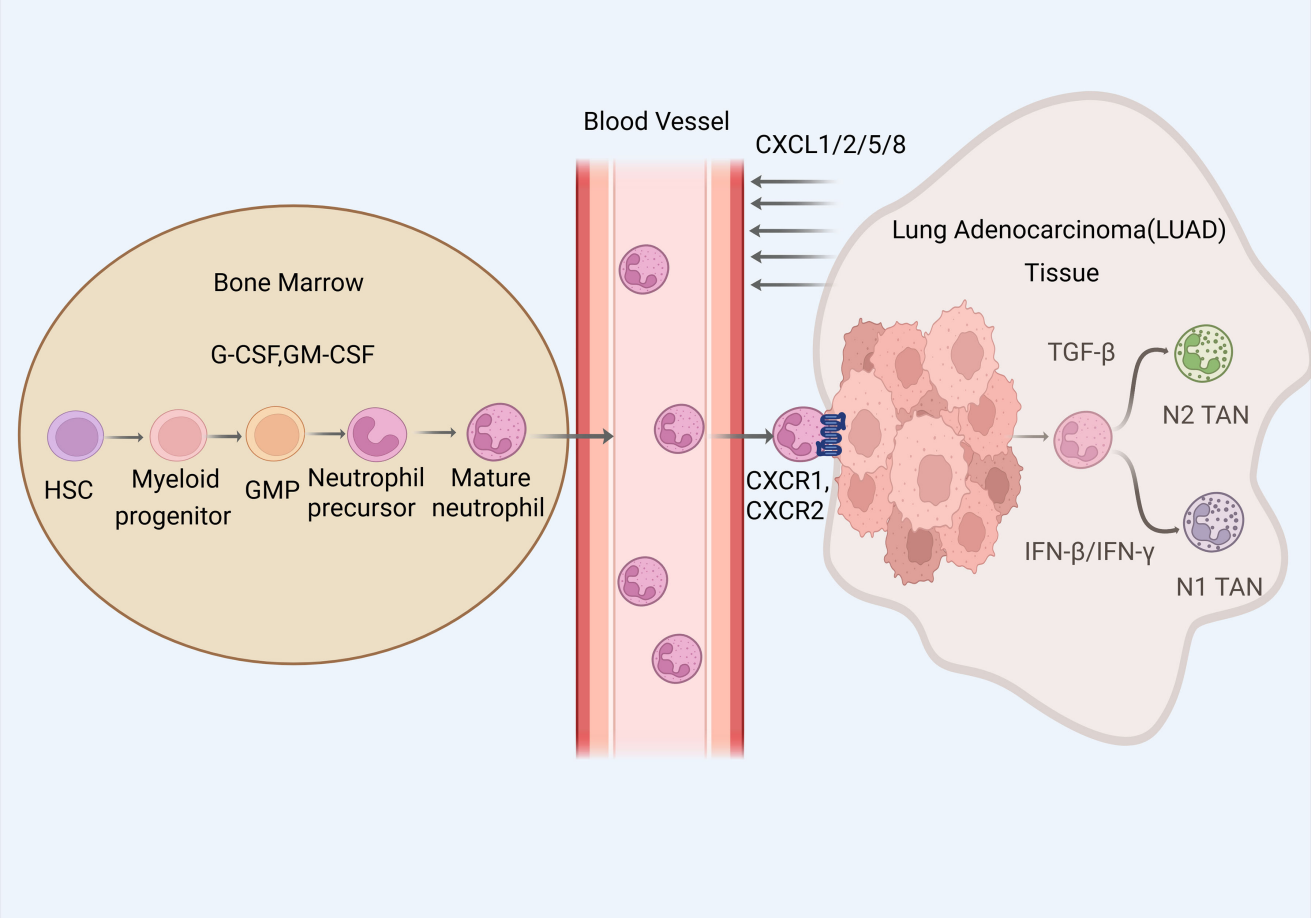
Origin, recruitment, and polarization of TANs in LUAD. (Schematic: bone marrow → CXCR1/2 axis → TME → TGF-β drives N2, IFN-β/γ drives N1).

TANs exhibit high plasticity and can be broadly classified into anti-tumorigenic N1-like and pro-tumorigenic N2-like phenotypes based on surface markers and functions (28). In this classic framework, N1-like TANs highly express TNF-α, ICAM-1, and CCL3 with low ARG1 expression, whereas N2-like TANs are characterized by high ARG1, CCL2, and vascular endothelial growth factor (VEGF) expression with downregulated TNF-α and ICAM-1 (12, 29). In the NSCLC (including LUAD) microenvironment, TGF-β is a key driver of N2 polarization, whereas interferon γ (IFN-γ) combined with Toll-like receptor (TLR) agonists promotes N1 polarization (12, 30). However, this classic framework is currently used more as a historical conceptual model. With the advancement of single-cell sequencing, it has been recognized that the N1/N2 dichotomy is an oversimplification. TANs exist along a continuous functional spectrum without well-defined lineage-specific markers akin to M1/M2 macrophages (13, 14). Therefore, more refined subpopulation classifications (e.g., immunomodulatory, pro-angiogenic, interferon-responsive subpopulations) are gradually replacing this simple binary model to more accurately describe the true heterogeneity of TANs (27). The above-described origin, recruitment and polarization of TANs in LUAD are summarized in **Figure 1**.

### 2.3 Spatial distribution of TANs in LUAD

The spatial distribution of TANs within tumor tissues exhibits significant heterogeneity and is associated with prognosis (28). In NSCLC (including LUAD), neutrophils infiltrate both the tumor core (nests) and invasive margins, as well as surrounding tertiary lymphoid structures (31, 32). Multiplex immunofluorescence and spatial transcriptomics studies indicate that neutrophils in the tumor stroma are more abundant in early stages (IA, IIA) and correlate with better prognosis, whereas intratumoral neutrophil infiltration in middle-to-late stages (IIB, IIIB) predicts poor prognosis (32, 33). Overall, in NSCLC, high-density CD66b+ TANs are generally associated with shorter disease-free survival (DFS) and overall survival (OS), a trend that holds true for the LUAD subtype, although this association is influenced by histological subtype, stage, and spatial location (13, 34). For example, in lung squamous cell carcinoma, TANs are more abundant and more pro-angiogenic, whereas LUAD exhibits more complex subpopulation compositions (13). Therefore, evaluating the prognostic value of TANs requires consideration of their density, spatial distribution, and molecular phenotype.

## 3 Dual functions of TANs in LUAD and their molecular mechanisms

### 3.1 Anti-tumor effects

TANs with anti-tumor phenotypes (often referred to as “N1-like” neutrophils) can inhibit LUAD progression through multiple mechanisms. First, they can directly kill tumor cells via antibody-dependent cellular cytotoxicity (ADCC). Neutrophils utilize their surface Fc receptors to recognize and adhere to antibody-coated cancer cells, releasing cytotoxic granular contents (myeloperoxidase, neutrophil elastase, matrix metalloproteinases) to induce non-apoptotic necrosis (12, 35). Furthermore, neutrophils can exert direct cytotoxicity via reactive oxygen species (ROS) and reactive nitrogen species (36). Recent single-cell studies have identified specific subpopulations with anti-tumor potential. For instance, an IL-8-driven CD74high neutrophil subpopulation in NSCLC models is capable of cross-presenting tumor antigens and directly activating CD8+ and CD4+ T cells, effectively suppressing tumor progression (37). These findings suggest that TANs can actively participate in anti-tumor immune responses under specific immune-activating conditions.

### 3.2 Pro-tumor effects

**Figure 2:**
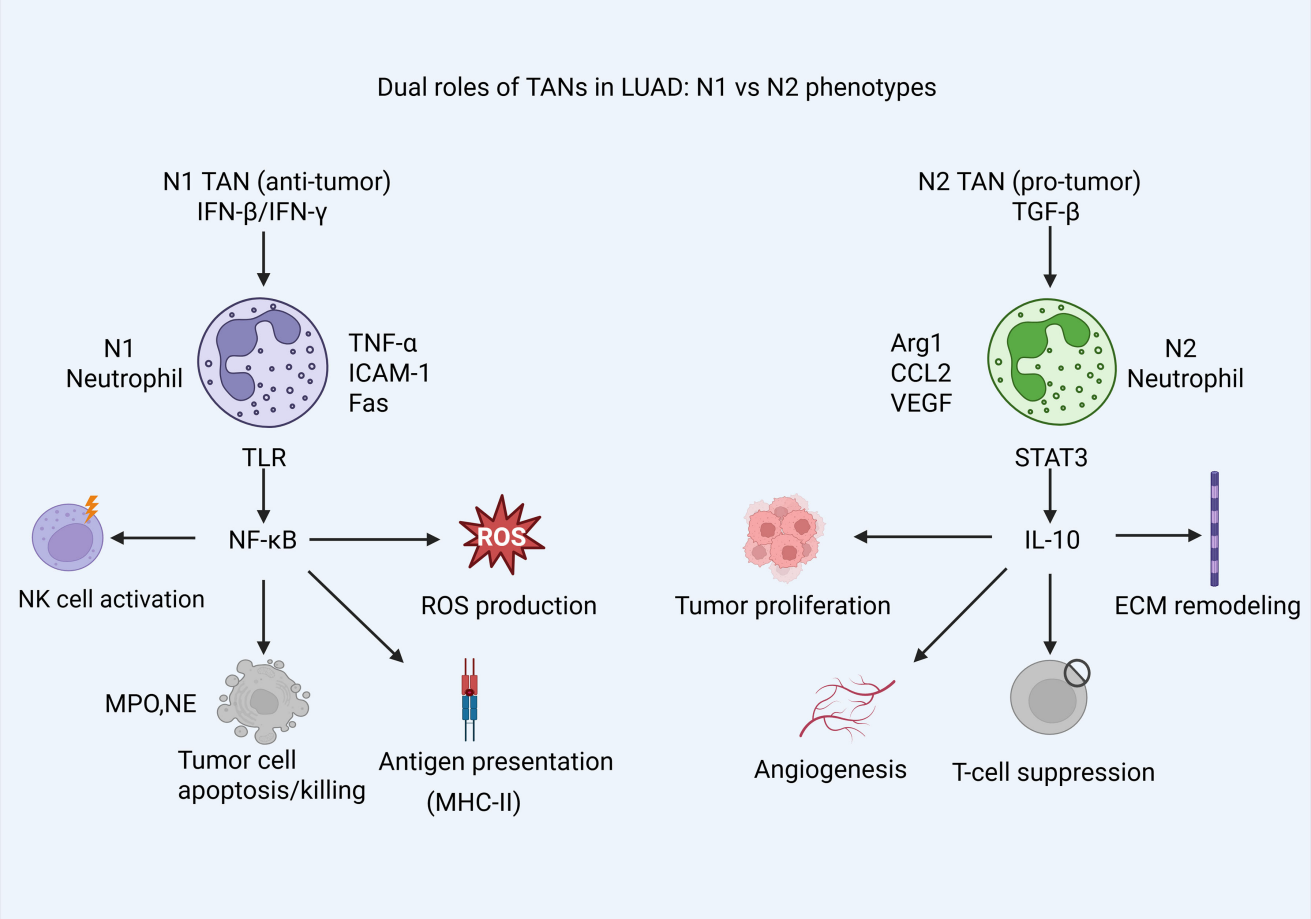
Dual functions of TANs in LUAD: N1 vs N2 pathways and downstream effectors.

Despite the existence of anti-tumor subpopulations, preclinical and clinical data indicate that TANs in NSCLC (including LUAD) predominantly exhibit pro-tumor effects, and high TAN infiltration is closely associated with poor patient prognosis (38). Under chronic stimulation within the TME, neutrophils can be skewed towards a pro-tumorigenic “N2-like” phenotype. These N2-like TANs promote tumor progression via multiple mechanisms: they secrete large amounts of ROS and proteases (e.g., MMP8/9) to enhance tumor cell invasiveness and degrade the extracellular matrix to pave the way for metastasis (12, 36). In terms of immunoregulation, N2-like TANs secrete ARG1 and inducible nitric oxide synthase (iNOS), depleting L-arginine in the microenvironment to inhibit T-cell proliferation and function (39). They can also express programmed death-ligand 1 (PD-L1), directly inducing CD8+ T-cell exhaustion (40). Additionally, TANs produce lipid mediators such as prostaglandin E2 (PGE2), further reinforcing the immunosuppressive microenvironment (41). It is necessary to clarify the relationship between TANs and polymorphonuclear myeloid-derived suppressor cells (PMN-MDSCs) when discussing the above pro-tumor mechanisms. TANs are defined as neutrophils present in the TME that are directly associated with tumor tissues. PMN-MDSCs are defined as low-density neutrophils with immunosuppressive functions, primarily found in the peripheral blood of cancer patients, pregnant women, or patients with infectious diseases (42). Studies have identified surface markers such as CD52, CD84, and PTGER2 that are highly expressed in PMN-MDSCs but are also enriched in TANs (42). This implies that these markers may be applicable for identifying both circulating PMN-MDSCs and TANs in the TME. In practice, distinguishing these two cell populations may depend on their source location: PMN-MDSCs are primarily isolated from peripheral blood, whereas TANs are obtained from tumor tissues (43). Notably, PMN-MDSCs in LUAD exhibit a high propensity for ferroptosis, and their high expression of insulin-like growth factor binding protein 1 positively correlates with poor prognosis, suggesting that neutrophil ferroptosis may represent a newly recognized immunosuppressive mechanism (29). The dual functions of TANs in LUAD discussed above are summarized in **Figure 2**.

### 3.3 Controversial role of NETosis in LUAD

**Figure 3:**
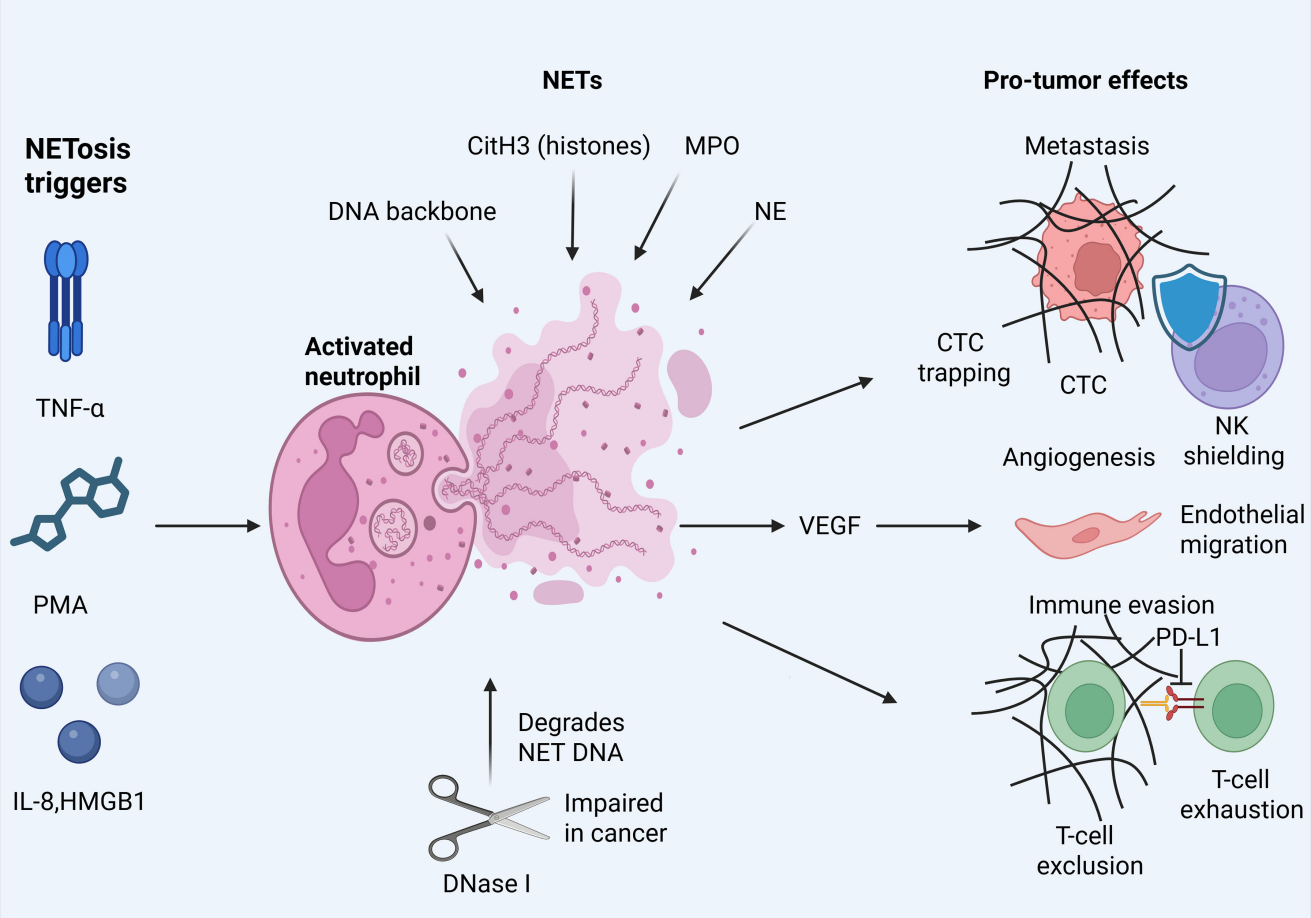
Formation of NETs and their pro tumor mechanisms in LUAD (including capture of CTCs, ECM degradation, CD8^+^ T cell exclusion).

Neutrophil extracellular traps (NETs) are web-like structures released by neutrophils upon specific stimulation, composed of decondensed chromatin DNA, histones, and granule proteins (neutrophil elastase, myeloperoxidase) (44). The role of NETosis in lung cancer (including LUAD) is dual and controversial. On the one hand, certain NET components (e.g., myeloperoxidase, histones) have direct tumor-killing potential, thereby suppressing tumor growth (45). On the other hand, NETs can serve as a source of antigens and adjuvants, taken up by dendritic cells and cross-presented to T cells, thereby initiating adaptive anti-tumor immunity (46, 47). It is worth noting that NETosis plays an irreplaceable role in host defense—it is the core mechanism by which neutrophils capture and kill pathogens such as bacteria and fungi (18, 48). Therefore, when considering anti-tumor strategies targeting NETs, the potential impact on normal anti-infective immunity must be weighed to avoid increasing infection risk due to excessive inhibition (49, 50). Nevertheless, under the continuous stimulation of the LUAD TME, NETs primarily promote tumor progression. Mechanistically, the physical DNA backbone of NETs can surround circulating tumor cells, promoting their adhesion to endothelial cells and subsequent distant metastasis (51). NETs also carry proteases such as neutrophil elastase and MMP9, which degrade the extracellular matrix and promote VEGF release, thereby accelerating angiogenesis and tumor invasion (51, 52). For instance, phospholipid scramblase 1 promotes LUAD progression by regulating NET formation, and its high expression is associated with poor patient prognosis (33). Furthermore, NETs stabilize SLC2A3 mRNA via m6A modification, inducing ferroptosis resistance in tumor cells while suppressing CD8+ T-cell function, thereby driving LUAD growth from multiple dimensions (53). The formation of NETs and their pro-tumor mechanisms in LUAD discussed above are summarized in **Figure 3**.

### 3.4 Interactions between TANs and T cells/macrophages

The functions of TANs are inextricably linked to their dynamic interactions with other immune cells within the TME. Among these, interactions with T cells and tumor-associated macrophages (TAMs) are particularly critical. Due to their DNA-rich scaffolding, NETs can form physical barriers that limit contact between CD8+ T cells and tumor cells, thereby reducing immune killing efficiency (54). Additionally, PD-L1 can be detected on the surface of NETs, directly leading to T-cell dysfunction and exhaustion (40). A complex bidirectional regulatory relationship exists between TANs and TAMs. Studies in NSCLC have shown that TANs recruit pro-tumorigenic SPP1+macrophages by secreting CCL3 and CCL4, which act on CCR1 and CCR5. In turn, SPP1+macrophages further promote the expansion of CCL3+ TANs via a positive feedback loop in patients unresponsive to immunotherapy, forming a vicious cycle that exacerbates the formation and maintenance of the immunosuppressive microenvironment (55).

## 4 Therapeutic strategies targeting TANs: preclinical evidence and translational bottlenecks

Given the critical role of TANs in LUAD progression and immunotherapy resistance, targeting TANs has become a key strategy to improve the efficacy of existing therapies. Current research directions include inhibiting TAN recruitment, blocking NET formation, modulating TAN polarization, and directly depleting TANs.

### 4.1 Inhibiting TAN recruitment

The recruitment of TANs to the TME primarily relies on chemokine receptor pathways, with the CXCR1/CXCR2 axis being particularly critical (26). Preclinical studies in NSCLC (including LUAD) mouse models have shown that CXCR1/CXCR2 antagonists (e.g., Reparixin, SX-682, Navarixin) effectively reduce intratumoral TAN infiltration and significantly enhance sensitivity to ICIs (26, 56). A phase II clinical trial of SX-682 combined with pembrolizumab in metastatic NSCLC is ongoing (NCT05570825), and its results are expected to provide clinical translational evidence. Additionally, strategies targeting upstream chemokines (e.g., the anti-CXCL8 antibody HuMax-IL8) have demonstrated safety in early-phase solid tumor clinical trials, though monotherapy efficacy remains limited (26). Recent studies have also identified other molecular mechanisms regulating TAN recruitment. The IGF2BP2 inhibitor JX5 significantly inhibits TAN recruitment by downregulating LOX1 expression (57). IL-37d inhibits early neutrophil migration to the lungs by suppressing TLR3 activation and S100A8/A9 expression in lung epithelial cells, thereby preventing the formation of pre-metastatic niches (58).

### 4.2 Inhibition of NETosis

Since NETs play a crucial role in promoting metastasis and immune evasion in lung cancer (including LUAD), targeting NETosis is a highly promising intervention. Preclinical studies have shown that PAD4 inhibitors (e.g., GSK484, Cl-amidine) can effectively block NET formation and reduce LUAD metastasis (29). DNase I can directly degrade the DNA backbone of NETs, and studies have demonstrated its ability to inhibit tumor progression and immune evasion in LUAD models (59). MEK inhibitors (e.g., selumetinib, trametinib) can reduce NET formation by blocking the Raf-MEK-ERK signaling pathway, inhibiting NADPH oxidase activity, and suppressing anti-apoptotic protein expression (60). Liraglutide reduces NETosis by inhibiting ROS production and enhances the anti-tumor efficacy of PD-1 inhibitors in preclinical models (61). However, NETosis plays a vital role in host anti-infective immunity, and long-term systemic inhibition may increase infection risk, constituting a major obstacle to clinical translation.

### 4.3 Modulation of TAN polarization

Given the functional plasticity of TANs, converting the pro-tumorigenic N2 phenotype to the anti-tumorigenic N1 phenotype is an attractive therapeutic concept. Targeting the TGF-β signaling pathway is a core strategy; in LUAD preclinical models, TGF-β inhibitors (e.g., galunisertib) in combination with ICIs successfully induced TAN polarization towards the N1 phenotype (12). Smad3, a key downstream effector of TGF-β signaling, can be inhibited by SIS3, which also promotes TAN polarization from N2 to N1 (62). Recent studies have revealed additional molecular targets that regulate TAN polarization. IL-8 has been found to induce the differentiation of a CD74high/SiglecFlow anti-tumor neutrophil subpopulation, and CD74 agonists can further enhance this effect (37). The IGF2BP2 inhibitor JX5 significantly suppresses N2 polarization by downregulating LOX1 expression (57). The PARP1 inhibitor AG14361 blocks the interaction between PARP1 and ALOX5 and their PARylation modification, inhibits MMP9 expression, and reverses the pro-tumor functions of neutrophils (63).

### 4.4 Depletion of TANs

Directly depleting TANs is a straightforward method to suppress their negative effects. In mouse models, anti-Ly6G antibodies can effectively deplete neutrophils and inhibit tumor growth (64). However, given the central role of neutrophils in anti-infective immunity, the clinical translation of this strategy faces severe safety challenges. Low-dose chemotherapy (e.g., paclitaxel) has been shown to selectively reduce N2-like TANs, though its specificity remains controversial (15). Notably, CCL21 dendritic cell (DC) *in situ* vaccination exhibits a unique advantage in depleting pro-tumorigenic neutrophil subpopulations by remodeling the immune landscape of the TME, significantly reducing the number of immunosuppressive neutrophils (65).

### 4.5 Combination therapeutic strategies

Given the functional complexity of TANs, single-target strategies often fail to achieve durable responses, making combination therapy the mainstream direction. Preclinical studies have confirmed that ICIs combined with CXCR2 inhibitors produce significant synergistic anti-tumor effects (56). Chemotherapy combined with NETosis inhibitors can effectively reduce chemotherapy-induced NET-associated metastasis. Although radiotherapy recruits neutrophils, its combination with TAN-modulating strategies (e.g., polarization modulators) requires further investigation. The therapeutic strategies targeting TANs discussed above are summarized in **Tables 1**, **2**.

**Table 1:** Therapeutic strategies targeting tumor-associated neutrophils (TANs) in lung adenocarcinoma (LUAD) and non-small cell lung cancer (NSCLC).

Strategy	Agent	Target/mechanism	Preclinical evidence (LUAD/NSCLC)	Clinical Trial Phase & ID (NSCLC)	Challenges for LUAD
Inhibition of TAN recruitment	SX-682	CXCR1/2 allosteric inhibitor	Reduced TAN infiltration; enhanced anti-PD-1 efficacy in murine LUSC model (1, 2)	Phase II: SX-682 + Pembrolizumab for metastatic NSCLC (NCT05570825)	Potential neutropenia risk; may compromise anti-infection immunity
Inhibition of TAN recruitment	Navarixin	CXCR1/2 allosteric inhibitor	Blocks CXCR1/2-dependent neutrophil recruitment (1)	Phase II: Navarixin + Pembrolizumab for advanced solid tumors including NSCLC (NCT03473925)	Limited efficacy as monotherapy; potential interference with beneficial neutrophil functions
Inhibition of TAN recruitment	HuMax-IL8 (BMS-986253)	Anti-IL-8 monoclonal antibody	Blocks IL-8; reverses immunosuppression in lung cancer models (1)	Phase II: Neoadjuvant Nivolumab + BMS-986253 for NSCLC/HCC (NCT04123379)	Weak monotherapeutic activity; prone to chemokine-mediated compensatory resistance; incomplete clearance of NETs
Inhibition of TAN recruitment	JX5	Small-molecule inhibitor targeting IGF2BP2 (m⁶A modification)	Inhibits m⁶A modification of LOX1, downregulates LOX1, suppresses TAN recruitment and N2 polarization (3)	Preclinical stage	Limited to preclinical data; functional limitations; failure to clear mature NETs
Inhibition of NETosis	Cl-amidine	Irreversible PAD4 inhibitor (blocks histone citrullination & NET formation)	Blocks NET formation; alleviates NET-associated damage in preclinical models (5)	Preclinical	Merely inhibiting NETosis yields limited effect on already formed NETs; off-target inflammation risk
Inhibition of NETosis	DNase I	NET-degrading enzyme (hydrolyzes DNA backbone)	Degrades NET DNA scaffold; inhibits tumor progression and immune evasion in LUAD models (51)	Preclinical	Short half-life; delivery challenges; may affect protective NETs against pathogens
Modulation of TAN polarization	Galunisertib (LY2157299)	TGF-βRI kinase inhibitor	Blocks TGF-β signaling; shifts TANs toward N1-like anti-tumor phenotype (7)	Phase I/II: Galunisertib + Nivolumab for advanced solid tumors including NSCLC (NCT02423343)	Broad target spectrum; limited monotherapeutic efficacy; potential cardiovascular toxicity
Modulation of TAN polarization	SIS3	Small-molecule Smad3 inhibitor	Blocks TGF-β/Smad3 signaling, inhibits N2 polarization (8)	Preclinical	Broad pharmacological activity with weak targeting specificity
Modulation of TAN polarization	AG14361	Selective PARP-1 inhibitor	Blocks PARP-1/ALOX5 interaction, suppresses MMP-9 expression, reverses pro-tumor neutrophil function (10)	Preclinical	High clinical toxicity risk; single mechanism predisposes to drug resistance
Depletion of TANs	CCL21-Gene Modified Dendritic Cell Vaccine	CCL21 binds CCR7; induces DC maturation & activation; eliminates pro-tumor neutrophils	Eliminates pro-tumor neutrophils; enriches type I interferon-responsive neutrophils (13)	Phase I: CCL21-DC vaccine + Pembrolizumab for stage IV NSCLC (NCT03546361)	Insufficient clinical data; limited routes of administration
Ancillary TAN/NET modulation (MEK inhibitors)	Selumetinib (AZD6244)	Non-ATP-competitive allosteric MEK1/2 inhibitor (ancillary reduction of NETs) (14)	Targeting Raf-MEK-ERK pathway suppresses NETs formation; indirect TAN-modulating effects	Phase II: AZD6244 + Docetaxel in KRAS mutation positive NSCLC (NCT01933932)	Broad-spectrum targeting induces off-target inflammation; not a specific NETosis inhibitor
Ancillary TAN/NET modulation (MEK inhibitors)	Trametinib (GSK1120212)	Reversible allosteric MEK1/2 inhibitor (ancillary effects on NETs) (14)	Targeting Raf-MEK-ERK signaling inhibits NETs formation; modulates TAN function	Phase II: Dabrafenib + Trametinib in BRAF V600E mutant metastatic NSCLC (NCT04452877)	Broad target spectrum leads to frequent off-target effects with poor targeting specificity

**Table 2:** Summary of Key Studies on Therapeutic Targeting of Tumor-Associated Neutrophils (TANs) in Lung Adenocarcinoma: Mechanisms, Translation, and Safety.

Category	Study / agent	Cancer type	Target	Mechanism	Model / patient cohort	Major findings	Translational relevance	Major safety issues
Recruitment Inhibition	SX-682 / Navarixin / Reparixin	NSCLC (including LUAD)	CXCR1 / CXCR2	Blockade of chemokine receptors, thereby inhibiting TAN recruitment into the tumor microenvironment (TME)	NSCLC mouse models; Phase II trial (NCT05570825)	Reduced intratumoral TAN infiltration and significantly enhanced sensitivity to immune checkpoint inhibitors (ICIs)	Phase II ongoing	Neutropenia, increased infection risk
Recruitment Inhibition	HuMax-IL8 (anti-CXCL8)	NSCLC (including LUAD)	CXCL8 (IL-8)	Targeting the upstream chemokine CXCL8 to reduce neutrophil recruitment	Early-phase solid tumor trials	Favorable safety profile, but limited monotherapy efficacy	Early phase	Not clearly defined; requires assessment in combination regimens
Recruitment / Polarization	JX5 (IGF2BP2 inhibitor)	LUAD	IGF2BP2 / LOX1	Downregulation of LOX1 expression, leading to suppression of TAN recruitment and N2 polarization	Preclinical (LUAD models)	Significantly inhibited TAN recruitment and blocked N2 polarization	Preclinical	To be determined (TBD)
Recruitment Inhibition	IL-37d	Lung cancer	TLR3 / S100A8/A9	Inhibiting TLR3 activation and S100A8/A9 expression to block early neutrophil migration	Preclinical (lung cancer models)	Prevented pre-metastatic niche formation and inhibited early pulmonary neutrophil migration	Preclinical	TBD
NETosis Inhibition	GSK484 / Cl-amidine	LUAD	PAD4	Inhibition of PAD4-mediated histone citrullination, blocking neutrophil extracellular trap (NET) formation	LUAD animal models	Effectively reduced LUAD metastasis	Preclinical	Increased infection risk (e.g., invasive aspergillosis)
NETosis Inhibition	DNase I	LUAD	NET DNA backbone	Direct degradation of the DNA backbone of NETs, disrupting NET structures	LUAD models	Inhibited tumor progression and immune evasion	Preclinical	Long-term use increases infection risk
NETosis Inhibition	Selumetinib / Trametinib	Lung cancer	MEK (Raf-MEK-ERK)	Blockade of the Raf-MEK-ERK signaling pathway, inhibiting NADPH oxidase and anti-apoptotic proteins	Preclinical (lung cancer models)	Reduced NET formation	Preclinical	TBD; potential impact on normal cell signaling
NETosis Inhibition	Liraglutide	Lung cancer (preclinical)	ROS pathway	Inhibition of reactive oxygen species (ROS) production, reducing NETosis	Preclinical models	Enhanced the anti-tumor efficacy of PD-1 inhibitors	Preclinical	TBD
Polarization Modulation	Galunisertib	LUAD	TGF-β	Blockade of TGF-β signaling, inducing TAN polarization toward the anti-tumorigenic N1 phenotype	LUAD preclinical models	Combination with ICIs successfully induced N1 polarization and augmented anti-tumor efficacy	Preclinical	Impacts tissue repair and immune homeostasis
Polarization Modulation	SIS3	LUAD	Smad3	Inhibition of Smad3, a downstream effector of TGF-β, thereby blocking N2 polarization signals	Preclinical (LUAD)	Promoted TAN conversion from the pro-tumorigenic N2 to the anti-tumorigenic N1 phenotype	Preclinical	TBD
Polarization Modulation	CD74 agonists	NSCLC	CD74	Induction of CD74^high^/SiglecF^low^ anti-tumor neutrophil subpopulation differentiation	Preclinical (NSCLC)	IL-8 induces this subpopulation; CD74 agonists further potentiate anti-tumor effects	Preclinical	TBD
Polarization Modulation	AG14361	Lung cancer	PARP1 / ALOX5	Blocking the interaction between PARP1 and ALOX5, inhibiting MMP9 expression	Preclinical (lung cancer)	Reversed the pro-tumor functions of neutrophils	Preclinical	TBD
Depletion	Anti-Ly6G antibody	Lung cancer	Ly6G	Direct global depletion of neutrophils	Mouse tumor models	Inhibited tumor growth	Preclinical	Severe infection risk; difficult clinical translation
Depletion	Low-dose paclitaxel	Lung cancer	N2-like TANs	Selective reduction of N2-like TANs (specificity remains controversial)	Preclinical (lung cancer)	Reduced N2-like TAN subpopulations	Preclinical (controversial)	Questionable specificity; potential impact on normal immunity
Depletion	CCL21 DC in situ vaccine	Lung cancer	Pro-tumor neutrophil subpopulations	Remodeling the TME immune landscape and depleting pro-tumorigenic neutrophils	Preclinical (lung cancer)	Significantly reduced pro-tumor neutrophil subpopulations	Preclinical	TBD
Metabolic Targeting	Numidargistat (ARG1 inhibitor)	LUAD	ARG1	Inhibition of ARG1, restoring L-arginine levels and relieving T-cell suppression	LUAD preclinical models	Restored T-cell activity and enhanced immunotherapy efficacy	Preclinical	TBD
Metabolic Targeting	Metformin / GLUT1 inhibitors / FATP2 inhibitors / IDO inhibitors	LUAD	GLUT1 / FATP2 / IDO, etc.	Targeting glucose uptake, fatty acid oxidation, tryptophan metabolism, etc., to remodel TAN metabolism	LUAD preclinical models	Limited the pro-tumor activity of TANs and enhanced conventional therapy and ICI efficacy	Preclinical	Off-target effects; risk of metabolic interference

Cancer type annotations: "LUAD" indicates lung adenocarcinoma-specific evidence; "NSCLC (including LUAD)" indicates studies in non-small cell lung cancer that include LUAD subgroups; "Lung cancer" refers to general pulmonary malignancy studies. Translational relevance: "Preclinical" refers to animal models or in vitro studies; "Early phase" indicates Phase I or exploratory clinical trials; "Phase II ongoing" indicates registered Phase II clinical trials.

## 5 Neutrophils as biomarkers in lung adenocarcinoma

### 5.1 Peripheral blood biomarkers

Among peripheral blood indices, the neutrophil-to-lymphocyte ratio (NLR) is the most widely studied and easily detectable marker. Multiple meta-analyses have consistently demonstrated that elevated pretreatment NLR is significantly associated with shorter progression-free survival (PFS) and overall survival (OS) in advanced NSCLC patients treated with ICIs, and it is currently primarily validated as a prognostic biomarker (66, 67). For instance, a meta-analysis of 1, 845 patients showed that the high NLR group had a significantly increased risk of death (HR: 2.50, 95% CI: 1.79–3.51) (68). Based on existing clinical evidence, baseline NLR should be routinely measured before initiating immunotherapy in NSCLC patients. When NLR ≥ 5, an immunochemotherapy combination regimen is directly recommended. If NLR remains elevated at week 6 of treatment, immediate imaging assessment and treatment adjustment are indicated; if NLR significantly decreases, continuation of the original immunotherapy is warranted (68). The derived NLR (dNLR) is also an independent poor prognostic marker and is closely associated with lower CD8+ T-cell infiltration density in tumor tissues (69, 70). The clinical translation pathway for peripheral blood dNLR as a practical biomarker for first-line pembrolizumab in NSCLC involves detecting dNLR levels (with a cutoff of 2.6) before treatment to rapidly screen the dominant population with high PD-L1 expression (TPS ≥ 50%) and dNLR < 2.6, for whom pembrolizumab monotherapy is prioritized to avoid the additional toxicity of chemoimmunotherapy. For patients with baseline dNLR ≥ 2.6, dynamic monitoring of dNLR changes at the second treatment cycle is recommended; an upward trend indicates primary resistance risk, necessitating a switch to a chemotherapy-containing combination regimen or exploration of intervention strategies targeting neutrophil-related immunosuppressive pathways, thereby enabling individualized treatment decisions and efficacy optimization (70). Beyond cell ratios, the concentration of soluble proteins in peripheral blood also holds prognostic value. Elevated serum CXCL8 (IL-8) and G-CSF levels are not only positively correlated with tumor burden but also associated with poor ICI efficacy and resistance (23, 26). These are currently primarily validated as prognostic markers, though their predictive potential is under investigation.

### 5.2 Tissue biomarkers

At the tissue level, the infiltration density and spatial distribution of intratumoral neutrophils (TANs) are key histopathological markers. Immunohistochemical detection of CD66b+ neutrophils in numerous studies has shown that higher TAN density is generally associated with poor prognosis in NSCLC patients, a finding that holds true in the LUAD subtype (32, 33). Specifically, in early-stage resectable NSCLC (including LUAD), high intratumoral CD66b+ neutrophil density is an independent predictor of recurrence beyond TNM staging and serves as a prognostic biomarker (71). The clear clinical translation pathway for CD66b+ neutrophils involves establishing a standardized multiplex immunohistochemistry detection and scoring system (key points include accurately distinguishing intratumoral versus stromal cells and setting >5% as the high-density threshold), validating its independent prognostic value in combination with TNM staging through multicenter prospective studies, and ultimately integrating CD66b+ neutrophil density as a key immune indicator into the TNM-immune scoring system for LUAD to provide a reliable basis for personalized treatment strategies (71). Beyond simple density calculation, the spatial relationships between TANs and effector cells provide more refined prognostic information. Studies have shown that the spatial distance between TANs and CD8+ T cells has significant predictive value; when the distance between TANs and CD20+ B cells or CD4+ T cells increases, it often predicts worse DFS (31, 72). This spatial separation may reflect an immune exclusion barrier formed by TANs, which hinders lymphocytes from exerting effective anti-tumor immunity (13). A spatial immune architecture quantification model measures the micro-distances between CD4+ T cells, CD20+ B cells, and neutrophils to construct a clinically applicable prognostic assessment system. This model confirms that when the distance between immune cells exceeds a specific threshold, the risk of postoperative recurrence increases by 2.7-fold. These spatial features can be directly translated into three categories of clinical decisions: identifying high-risk populations requiring intensified adjuvant therapy, warning patients for whom immunotherapy monotherapy may fail, and guiding the formulation of combination treatment regimens, ultimately achieving a complete transformation from laboratory observation indicators to clinical treatment standards (31). Furthermore, specific TAN subpopulations identified through multiplex immunofluorescence and single-cell sequencing (e.g., immunosuppressive LOX1+ or PD-L1+ subpopulations), when present at high abundance, also predict poor response to immunotherapy; current evidence primarily supports their prognostic value, with some studies suggesting their predictive potential for ICI response (27, 30).

### 5.3 NET components in liquid biopsy

NET components as liquid biopsy markers have become a research hotspot. Unlike the peripheral blood cell ratios and tissue markers discussed earlier (which are mostly baseline biomarkers measured once before treatment), the dynamic changes in NET components during treatment endow them with unique value. In LUAD patients, significantly elevated NET-related degradation products, such as citrullinated histone H3 (CitH3), myeloperoxidase-DNA complexes, and circulating free DNA, can be detected in peripheral blood. Studies have shown that elevated plasma NET marker levels are associated with an increased risk of distant metastasis and poorer prognosis in LUAD patients (73, 74). Dynamic monitoring reveals that patients with persistently high or rising plasma CitH3 levels during immunotherapy have significantly inferior PFS and OS compared to those with declining CitH3 levels. A plasma CitH3 concentration >7.04 ng/mL is an independent risk factor for immunotherapy prognosis, corresponding to a 70.2% increased risk of death and a 56.6% increased risk of disease progression (75). This quantitative indicator provides an objective basis for clinical prognostic assessment and helps identify high-risk populations for recurrence and optimize treatment strategies. NET markers such as CitH3, distinct from baseline prognostic markers measured once before treatment, are more suitable as dynamic treatment monitoring markers for pharmacodynamic assessment during therapy and early warning of resistance. This finding offers a new perspective for non-invasive dynamic monitoring of the tumor immune microenvironment status.

### 5.4 Comparative analysis of biomarkers

The aforementioned markers can be categorized by source into peripheral blood and tissue types, each with distinct predictive performance and application scenarios. Peripheral blood markers (NLR, dNLR, CXCL8, sLOX-1, CitH3) share the advantages of being non-invasive, repeatable, and cost-effective, suitable for large-scale screening and dynamic monitoring. Among them, NLR/dNLR have the highest level of evidence and the most clearly defined clinical thresholds, making them the most practical baseline prognostic markers; CXCL8 and sLOX-1 offer superior mechanistic specificity but lack standardized detection; CitH3’s unique value lies in its ability to warn of resistance through dynamic monitoring during treatment, though its baseline predictive performance is weaker than NLR. Tissue markers (CD66b density, TAN-lymphocyte spatial distance) offer higher specificity and provide local immune microenvironment information that peripheral blood cannot obtain. Among these, the predictive performance of spatial distance is superior to simple density, but both rely on invasive sampling and advanced detection platforms, limiting accessibility and precluding dynamic monitoring. Therefore, no single marker can meet all clinical needs. Peripheral blood markers are suitable for first-line screening and treatment monitoring, while tissue markers are suitable for precise prognostic assessment. The two types are complementary rather than substitutable, and future efforts should explore multi-marker combination models.

## 6 Metabolic reprogramming of TANs and Its therapeutic potential

The metabolic state of immune cells is closely linked to their function, a concept particularly critical in the LUAD microenvironment. The tumor microenvironment (TME) of LUAD is characterized by hypoxia, nutrient deprivation, and metabolic waste accumulation resulting from rapid tumor cell proliferation, which drives infiltrating TANs to undergo profound metabolic reprogramming to sustain their survival and immunosuppressive functions (25). Unlike neutrophils in normal tissues, the metabolic patterns of TANs are distorted by tumor signals, forming a vicious cycle that promotes, rather than eradicates, tumor progression. A deeper understanding of these metabolic changes holds promise for opening up highly promising new strategies for LUAD treatment.

### 6.1 Glucose metabolic reprogramming

In terms of glucose metabolism, the LUAD TME is flooded with lactate produced by tumor cells and immune cells (76). To adapt to this environment, TANs significantly upregulate the expression of glucose transporter 1 (GLUT1), leading to a substantial increase in glucose uptake and glycolysis rates (77). This enhanced aerobic glycolysis not only provides energy for the rapid effector functions of TANs but also exacerbates the immunosuppressive properties of the TME through lactate accumulation. Studies have shown that lactate can induce PD-L1 expression on the surface of neutrophils, thereby inhibiting T-cell anti-tumor activity (25). Furthermore, although enhanced glycolysis is a primary feature, TANs also exhibit metabolic flexibility, utilizing fatty acid oxidation (FAO) and oxidative phosphorylation (OXPHOS) to sustain high migratory capacity and immunosuppressive functions, which is closely associated with poor clinical prognosis (78). Therefore, targeting GLUT1 or the glycolytic pathway, for example, by inhibiting glucose uptake to limit metabolic substrate supply or using drugs such as metformin to modulate mitochondrial respiration, has been shown to restrict the pro-tumor activity of TANs and enhance the efficacy of conventional therapies (25).

### 6.2 Lipid metabolic reprogramming

Lipid metabolic reprogramming is also a key step for TANs to acquire pro-tumor functions. In the LUAD TME, granulocyte-macrophage colony-stimulating factor (GM-CSF) upregulates the expression of fatty acid transport protein 2 (FATP2) in TANs via activation of STAT5 signaling (41). FATP2 mediates the uptake of arachidonic acid and promotes its conversion into immunosuppressive prostaglandin E2 (PGE2), a crucial mechanism by which TANs suppress CD8+ T-cell function (41). Additionally, TANs highly express lectin-type oxidized low-density lipoprotein receptor 1 (LOX-1), which promotes the uptake of oxidized low-density lipoprotein and participates in maintaining the immunosuppressive functions of TANs (79). In LUAD, the PPARγ signaling pathway has been reported to regulate lipid metabolic crosstalk, promoting cholesterol uptake by tumor cells and thereby driving tumor growth (25, 80). Targeted inhibition of FAO or FATP2 has been shown in preclinical models to attenuate the immunosuppressive capacity of TANs and inhibit tumor growth (25, 81).

### 6.3 Amino acid metabolic reprogramming

At the level of amino acid metabolism, arginine metabolism is a critical pathway through which TANs exert T-cell suppression. In LUAD, tumor-derived ANXA2 signaling induces high expression of arginase 1 (ARG1) in TANs via the TLR2/MYD88 axis (39). The secretion of ARG1 severely restricts the proliferation and effector functions of T cells by depleting L-arginine in the TME, thereby promoting immune evasion (39). Additionally, IL-8 stimulation can also induce the release of ARG1 from neutrophils (82). The use of ARG1 inhibitors (e.g., Numidargistat) can restore T-cell activity and has shown potential to enhance immunotherapy in preclinical models (83). Glutamine and tryptophan metabolism, and their roles as energy sources and metabolic messengers in TAN survival and immunosuppression, have also attracted widespread attention. For example, activation of the indoleamine 2, 3-dioxygenase (IDO) pathway can lead to tryptophan depletion, thereby exacerbating the immunosuppressive state of the microenvironment (25).

**Figure 4:**
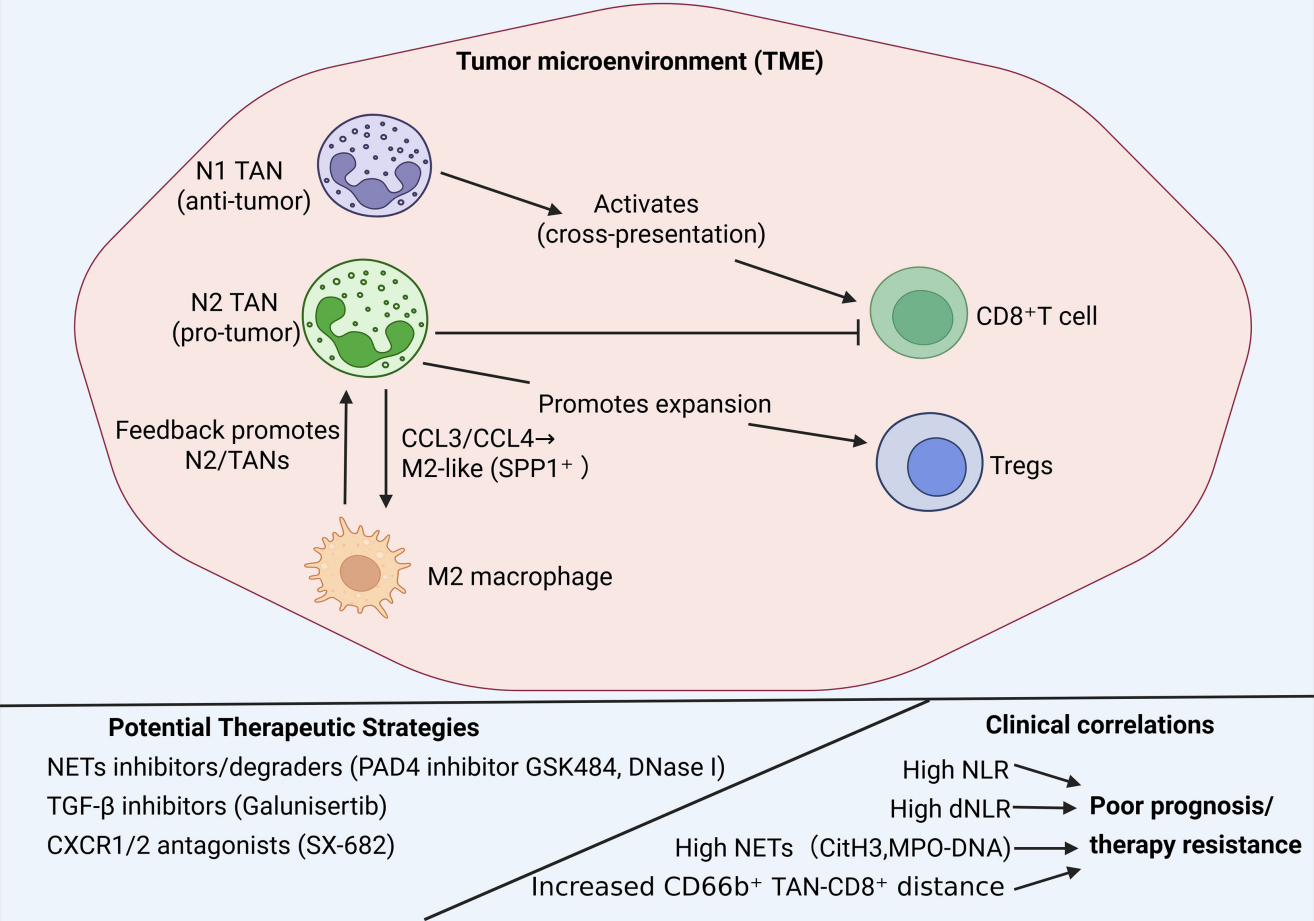
Interaction network of TANs with immune cells in LUAD and associated biomarkers/therapeutic interventions.

These metabolic pathways do not operate in isolation but are tightly intertwined, collectively supporting the pro-tumor phenotype of TANs. In terms of clinical therapeutic strategies, targeting these metabolic nodes holds direct translational significance. For instance, drugs such as metformin, GLUT1 inhibitors, FATP2 inhibitors, and IDO inhibitors are currently being investigated to “starve” or “reprogram” these immune cells hijacked within the TME, aiming to reverse their pro-tumor properties and enhance the efficacy of existing ICIs (25). Future research needs to further elucidate the specificity of these metabolic pathways across different TAN subpopulations and integrate multi-omics technologies to develop more precise metabolic-targeted combination therapeutic strategies, offering new avenues to improve the prognosis of LUAD patients. The interaction network of TANs with immune cells in LUAD and associated biomarkers/therapeutic interventions discussed above are summarized in **Figure 4**.

## 7 Discussion

In lung adenocarcinoma (LUAD), TANs exhibit significant functional heterogeneity and spatial distribution-dependent effects, with their pro-tumor and anti-tumor functions coexisting, profoundly influencing disease progression and treatment response. The core mechanisms of action are as follows: NSCLC (including LUAD) tumor cells and stromal cells abundantly secrete chemokines such as CXCL1, CXCL5, and CXCL8, which efficiently recruit circulating neutrophils to the TME via the CXCR1/CXCR2 axis (24, 26). Within the TME, signals such as TGF-β tend to drive TAN polarization towards the historically defined pro-tumorigenic N2 phenotype (12). Meanwhile, neutrophils can undergo NETosis, forming NETs rich in DNA and granule proteins (44). Polarized N2-like TANs and NETs synergistically shape the immunosuppressive microenvironment: they secrete ARG1 and other substances to deplete amino acids required for T-cell activation (39); directly express PD-L1 to induce T-cell exhaustion (40); and form physical barriers via NETs to hinder the contact and killing of tumor cells by CD8+ T cells (54). Furthermore, NETs encapsulate circulating tumor cells to promote distant metastasis (84) and degrade the extracellular matrix while promoting angiogenesis, thereby accelerating tumor progression (44). Collectively, these mechanisms weaken anti-tumor immune surveillance, ultimately leading to primary or acquired resistance to ICIs. This complex regulatory network highlights the value of TANs as key therapeutic targets and identifies three critical intervention windows: blocking CXCR1/2-mediated TAN recruitment at the “entry point”; reversing the N2 phenotype to the anti-tumorigenic N1 phenotype using TGF-β inhibitors “internally”; and degrading NETs using PAD4 inhibitors or DNase I at the “endpoint” to break the pro-metastatic and immunosuppressive barriers.

The clinical translation of TAN- and NET-targeted therapies faces several key obstacles, among which the most significant constraint is the potential impairment of host anti-infective immunity due to long-term suppression of neutrophil function or NET formation—this is the core safety hurdle that determines whether such strategies can enter long-term clinical application. Neutrophils are the first line of defense against bacteria (particularly *Pseudomonas aeruginosa*) and invasive fungi (e.g., *Aspergillus fumigatus*) (49, 50). CXCR2 is crucial for the mobilization of normal neutrophils from the bone marrow and their homing to inflammatory sites. Long-term application of CXCR1/CXCR2 inhibitors (SX-682, Reparixin, Navarixin) can lead to neutropenia, compromising the host’s defense against pathogens and thereby increasing infection risk (26, 56). Neutrophil depletion via anti-Ly6G antibodies similarly weakens physiological anti-infective capacity (64). NETosis is a core mechanism for capturing and killing extracellular pathogens; systemic PAD4 inhibition or long-term DNase I treatment has been shown in animal models to increase the risk of invasive aspergillosis and refractory *Pseudomonas aeruginosa* pneumonia (49, 50). Although TGF-β inhibitors (e.g., galunisertib) function by reprogramming polarization, off-target toxicity also exists due to their impact on tissue repair and immune homeostasis (12).

In addition to the aforementioned infection-related safety obstacles, current strategies suffer from two main limitations. First, the lack of specific markers to distinguish pathogenic TANs from physiological neutrophils. CXCR1/CXCR2 inhibitors, anti-Ly6G depletion, and TGF-β polarization modulators all act on global neutrophil function or numbers, making it difficult to selectively target pro-tumorigenic subpopulations within the tumor. Second, the high spatiotemporal heterogeneity and plasticity of TAN function. Within the same patient, different tumor regions, disease stages, and treatment lines may exhibit distinct TAN subpopulation compositions and dominant functions (13, 34). Therefore, treatment regimens must be dynamically adjustable. Additionally, combining TAN-targeted drugs with ICIs, chemotherapy, or radiotherapy, while potentially synergistic, may also introduce or exacerbate new toxicities.

Regarding the common dilemma in current TAN-targeted clinical trials—”significant preclinical efficacy but mediocre clinical results”—we believe that, in addition to the aforementioned issues of target specificity, safety challenges, and general translational difficulties, several key factors contribute to this translational gap that warrant attention in future research. First, the lack of biomarker-based patient stratification is a core reason for unsatisfactory trial results. Many early-phase trials did not screen patients for TAN-related biomarkers. For example, high pretreatment NLR is significantly associated with poor ICI efficacy (85); high CXCL2 expression in LUAD (86), high intratumoral CD66b+ TAN density (71), specific pro-tumorigenic subpopulations (LOX1+ or PD-L1+TANs) (27, 30), or elevated plasma CXCL8 (IL-8) (23), sLOX1 (87), and NET markers (CitH3) (74) all predict poor prognosis and potential treatment resistance. Without stratifying patients based on these markers, efficacy signals are easily diluted by heterogeneous populations. Second, the timing and strategy of combination therapy have not been optimized. Preclinical studies have confirmed synergy between CXCR2 inhibitors and PD-1/PD-L1 inhibitors (56), but in clinical practice, the optimal administration sequence (e.g., administering TAN-targeted drugs first to remodel the TME, or concomitant administration) remains unclear. A rational hypothesis is a “clear first, then build” sequential approach—i.e., initially using CXCR2 antagonists to clear pro-tumor TANs and degrade the NET barrier, followed by the administration of ICIs to activate T cells—which may yield optimal synergistic effects. However, most current clinical trials employ concomitant dosing regimens, which may not maximize synergy and could even limit dose intensity due to overlapping toxicities. Finally, existing drugs may not achieve sufficient, thorough, or durable modulation of TANs. For example, although CXCR1/CXCR2 inhibitors can block neutrophil recruitment (26), they cannot eliminate already infiltrated and functionally polarized TANs, and have limited effects on existing NETs. Similarly, due to infection risks, direct depletion strategies or NETosis inhibitors may be forced to use lower doses or shorter courses in clinical trials, failing to achieve complete suppression. Moreover, TANs exhibit high plasticity and redundant recruitment pathways; when a single target is inhibited, tumors may compensate through other chemokine axes (e.g., CCL2-CCR2) (88).

As mentioned earlier, the traditional N1/N2 dichotomy serves as a heuristic historical conceptual framework but is insufficient to fully capture the high heterogeneity and functional plasticity of TANs in LUAD (13). Emerging technologies such as single-cell transcriptomics, high-dimensional flow cytometry, and spatial multi-omics have revealed that TANs form a continuous spectrum comprising multiple functionally distinct subpopulations (27). Identifying these novel subpopulations has significant clinical implications. Interferon-responsive neutrophils are characterized by high expression of interferon-stimulated genes. Under acute, robust interferon signaling (e.g., effective immunotherapy), they exert anti-tumor effects by activating CD8+ T cells and inhibiting angiogenesis to eliminate tumors; conversely, under chronic, weak interferon conditions (e.g., chronic inflammation or advanced cancer), they may switch to a pro-tumor phenotype, accelerating disease progression by sustaining detrimental inflammation or promoting cell survival (27). Historically considered “hybrid” cells primarily present in early-stage tumors and absent in advanced stages, recent studies have revealed that a CD74high neutrophil subpopulation driven by IL-8 also possesses antigen-presenting capacity in advanced NSCLC. Rather than being absent, their presence is closely associated with optimal responses to ICIs and prolonged survival, serving as a key positive predictive factor for efficacy (27, 37). Pro-angiogenic neutrophils are a critical pro-tumorigenic subpopulation of TANs characterized by high expression of pro-angiogenic factors such as VEGFA. They are primarily driven by tumor hypoxia and directly drive tumor growth and dissemination by promoting new vessel formation and providing a “bed” for metastasis of circulating tumor cells (27). LOX-1+ suppressive neutrophils are a key marker population of PMN-MDSCs in human cancers. They directly suppress the function of cytotoxic T cells by releasing immunosuppressive signals (e.g., ROS), thereby driving tumor immune evasion. In the hypoxic microenvironment of lung cancer, LOX-1+ cells undergo lipid metabolic reprogramming by binding ox-LDL, upregulating PD-L1 expression to enhance immunosuppression; their accumulation is regulated by tumor-derived factors such as CXCL5, and plasma sLOX-1 levels are positively correlated with tumor burden, making them a dynamic biomarker reflecting the microenvironmental state (27, 29). PD-L1+ neutrophils represent a functional state rather than a fixed subtype, characterized by the expression of the immune checkpoint PD-L1. They mediate immunosuppression by directly inhibiting the anti-tumor activity of T cells via the PD-1/PD-L1 pathway. In the lung cancer microenvironment, tumor cells induce PD-L1 expression by secreting CXCL5/GM-CSF to activate the PXN/AKT pathway, while metabolic stress such as ferroptosis can also upregulate PD-L1 levels. This suppressive phenotype dynamically intensifies with tumor progression, reflecting the precise regulation of their function by microenvironmental signals (27, 29). Compared with traditional indicators such as total TAN density or peripheral blood NLR, the abundance of these specific subpopulations can more accurately predict patient prognosis and ICI response. Therefore, shifting from simple quantitative analysis to subpopulation-specific molecular definition-based typing is crucial for precision immunotherapy. Future research should focus on constructing a multi-dimensional TAN typing system that integrates functional status, spatial localization, and metabolic characteristics. This includes not only determining their secretory profiles and surface receptors but also elucidating their spatial distribution across different micro-regions (tumor core, invasive front, tertiary lymphoid structures), as TANs located in different regions may have opposing functions (32, 33). Additionally, the specific metabolic states of TANs, such as arginine depletion and susceptibility to ferroptosis (29, 39), should be incorporated into the typing system. The ultimate goal is to map a multi-dimensional “functional-spatial-metabolic” atlas to identify key “harmful” subpopulations driving disease progression as therapeutic targets, while preserving or enhancing “beneficial” subpopulations with anti-tumor potential. This in-depth understanding will guide the development of drugs targeting specific subpopulations (e.g., anti-LOX1 antibodies) and help screen patients most likely to benefit from neutrophil-targeted combination therapy strategies.

To achieve clinical translation of TAN-targeted therapies in locally advanced LUAD, future research should focus on developing intervention strategies with spatiotemporal precision and functional specificity. One major research direction is the development of dynamic visualization tools and precision targeting therapies. Molecular imaging probes based on TAN-specific markers (e.g., CXCR2) or core NET components (e.g., CitH3) can enable non-invasive *in vivo* monitoring of TAN/NET dynamics, thereby guiding therapeutic timing. Meanwhile, pro-tumorigenic subpopulation-specific surface markers identified through single-cell omics (e.g., LOX1) can be used to design antibody-drug conjugates (ADCs), bispecific antibodies, or TME-sensitive nano-delivery systems to selectively eliminate or remodel harmful TANs, maximizing anti-tumor efficacy while preserving normal host immune defense functions. Another key direction is intervening in the pathogenic interaction networks between TANs and other TME cells. For example, targeting the positive feedback loop in which TANs recruit SPP1+ macrophages via CCL3/4 (CCL3/4-CCR1/5 axis), or newly identified regulatory nodes (e.g., IGF2BP2-LOX1 axis, PARP1-ALOX5 pathway), may systematically break the synergistic immunosuppressive effects among myeloid cells and provide new targets for combination therapy.

Specific translational research could focus on optimizing combination therapy regimens. A rational hypothesis is a “clear first, then build” sequential approach—initially using CXCR2 antagonists (e.g., SX-682) to clear pro-tumor TANs and degrade the NET barrier, remodeling the immunosuppressive microenvironment, followed by the application of PD-1 inhibitors to activate T cells, which may yield optimal synergistic effects. However, the ongoing phase II clinical trial (e.g., NCT05570825, SX-682 With Pembrolizumab) employs a concomitant dosing regimen. This may be due to the need to simplify trial procedures, avoid delays associated with sequential therapy, and preliminary observations of synergy in preclinical models. Nevertheless, concomitant dosing may not maximize the depth and sustainability of TME remodeling and may even limit the respective dose intensities due to overlapping toxicities (e.g., neutropenia). In contrast, the “clear first, then build” sequential strategy is theoretically more attractive, but its safety and efficacy require careful evaluation. A key safety concern is that prior use of CXCR2 inhibitors may exacerbate or prolong neutropenia, thereby increasing infection risk before subsequent PD-1 inhibitor administration. To address this challenge, future clinical trial designs could consider the following strategies: first, using a lower starting dose of CXCR2 inhibitors during the “clear” phase to mitigate bone marrow suppression; second, setting an appropriate washout period between administrations to allow neutrophil count recovery; third, prophylactic use of granulocyte colony-stimulating factor (G-CSF) support if necessary in the sequential regimen. Furthermore, the theoretical advantages of this sequential strategy may be more pronounced in specific clinical scenarios. For example, in the neoadjuvant setting for resectable early-stage LUAD, patients typically have a relatively ample treatment window, allowing the “clear” phase (e.g., two to three weeks of CXCR2 inhibitor monotherapy induction) without delaying curative surgery. In this context, the long-term benefits of fully remodeling the TME may far outweigh the risks of short-term waiting. Conversely, for advanced or metastatic patients with high tumor burden and rapid progression, any treatment delay may risk disease progression, making concomitant dosing or biomarker-based rapid response screening strategies more pragmatic choices. Ultimately, whether concomitant or sequential dosing achieves the optimal balance between efficacy and safety awaits answers from specifically designed randomized controlled phase II trials that compare the pharmacodynamics, toxicity profiles, and clinical outcomes of the two regimens, with stratified analysis by disease stage.

In conclusion, within the complex and dynamic TME of LUAD, neutrophils are no longer merely short-lived inflammatory cells but play multiple, sometimes contradictory, roles: they are innate immune effector cells with direct tumor-killing potential; they are “hijacked” accomplices that promote immunosuppression, angiogenesis, and metastasis through polarization and NETosis; and they are also key obstacles to the efficacy of current ICIs. However, it is precisely this functional plasticity and mechanistic complexity that make neutrophils highly promising targets for intervention and sources of predictive biomarkers. In the future, with deeper application of single-cell and spatial multi-omics technologies and further elucidation of the regulatory networks governing TAN heterogeneity, we believe that strategies targeting neutrophils will evolve from “indiscriminate suppression” to “precise modulation and remodeling.” By blocking recruitment, reversing polarization, depleting harmful subpopulations, or degrading pro-metastatic NETs, and combining these approaches with existing immunotherapies and chemotherapy in a spatiotemporally sequential manner, this traditionally perceived “therapeutic obstacle” is expected to transform into a new breakthrough to overcome LUAD treatment challenges, ultimately improving clinical outcomes for patients.
